# Facile Fabrication of Biochar from Palm Kernel Shell Waste and Its Novel Application to Magnesium-Based Materials for Hydrogen Storage

**DOI:** 10.3390/ma13030625

**Published:** 2020-01-31

**Authors:** Martin Luther Yeboah, Xinyuan Li, Shixue Zhou

**Affiliations:** 1 College of Chemical and Environmental Engineering, Shandong University of Science and Technology, Qingdao 266590, China; m13210013249@163.com (M.L.Y.); lixinyuan@163.com (X.L.); 2 State Key Laboratory of Mining Disaster Prevention and Control Co-founded by Shandong Province and The Ministry of Science and Technology, Shandong University of Science and Technology, Qingdao 266590, China

**Keywords:** magnesium, palm kernel shell biochar, hydrogen storage

## Abstract

In this investigation, an easily-operated and cost-effective method is utilized to synthesize biochar in ambient air, and the prepared biochar is used in a novel manner as a milling aid for fabricating Mg-biochar composites for hydrogen storage. X-ray diffractometry reveals that increasing the content of palm kernel shell biochar (PKSBC) from 5 wt.% to 20 wt.% enhances the hydrogen absorption performance by increasing the conversion of Mg into MgH_2_ from 83% to 93%. A 40 °C reduction in decomposition temperature of MgH_2_ is recorded from differential scanning calorimetry curves when the content of PKSBC is increased to 20 wt.%. Magnesium is milled and hydrided under the same experimental conditions and used as a reference material. It is proposed that these property enhancements can be attributed to the fact that PKSBC acts as an anti-sticking agent for elemental Mg powders, helping in the achievement of a more dispersed composite with reduced Mg particle size due to its layered-like carbon structure.

## 1. Introduction

Magnesium is considered a potential contender for hydrogen storage due to its high theoretical hydrogen storage capacity (7.6 wt.%) [1], abundance, and low cost. Despite this, several hindrances limit the application of magnesium as a hydrogen storage material. Magnesium reacts with hydrogen at high temperature (> 350 °C) and high-pressure conditions to form a thermodynamically stable hydride (high enthalpy change of hydrogen desorption of 74.6 kJ/mol [2]) which possesses a low hydrogen sorption rate [3,4]. In the quest to improve upon the sorption kinetics of magnesium-based hydrogen materials, various property-enhancing techniques such as nanoconfinement [5], catalytic metals [6,7,8,9], and catalytic metal oxide [10,11,12,13,14] addition, carbon incorporation [15], and synergistic catalysis [16] have been employed. Carbonaceous materials such as carbon black [17], graphite [18,19], carbon nanotubes [20], and crystallitic carbon from coal [21,22] have shown tremendous usefulness in improving the sorption properties of magnesium through ball milling. Downsizing, via ball milling, is another technique that has been employed to control the particle size of magnesium as well as introduce numerous defects in the structure of magnesium for enhanced sorption properties. Magnesium is soft and easily agglomerates into a big particle during ball milling. In order to achieve nanosized Mg/MgH_2_ particle sizes during mechanical ball milling, dispersing agents, and milling aids are utilized [21]. 

Imamura et al. [23,24,25], in a series of works, employed liquid organic milling aids (i.e., cyclohexane and benzene) and other carbon-containing solid materials [17,26,27] with dispersive properties to produce nanosized Mg/MgH_2_ particles after long hours of high energy ball milling. However, carbon materials utilized as milling aids, lubricants, and dispersants are prepared by sophisticated production methods such as chemical vapor deposition, laser ablation, and plasma touch techniques. Also, precursors such as petroleum coke, coal, and pitch used for preparing these milling aids or additives are not environmentally friendly and are non-renewable, and there is therefore a need to explore new alternatives such as biomass and other waste materials. 

Oil palm is considered the second most crucial tropical plant in Ghana after cocoa, and its production is a significant pillar of Ghana’s agricultural sector. From the president’s unique initiative on oil palm in 2002, palm oil production was selected by the government as a critical strategic pillar of agricultural growth and reduction of poverty because it has a high possibility of providing income for many rural small-scale farmers. In 2009, Ghana produced about 2,103,600 metric tons (MT) of oil palm fruit bunches and 130,000 MT of palm oil. Oil palm fruit consists of a mesocarp, a shell, and a kernel. Palm kernel shell (PKS), the non-edible part of the palm fruit, is considered a waste material, and even though new ways of utilizing these waste materials have surfaced, its production outweighs its consumption. Hence, environmentally friendly methods of eliminating these palm kernel shells are of utmost importance to the environment.

Biochar, a carbon-rich, non-volatile solid residue product obtained from the carbonization of biomass or agricultural waste materials in the presence of little to no air, has been used in various applications such as fertilizers [28], dye colorant, and harmful chemical absorbent [29,30,31,32], as well as soil conditioner [33]. Its relevance with regard to preparing Mg/C composite for hydrogen storage is rarely reported. In this work we propose a simple, cost-effective, and safe method of preparing biochar in the absence of inert gas. Secondly, we use in a novel manner the prepared biochar as an additive into the ball milling process to produce Mg/C composites for hydrogen storage. 

## 2. Materials and Methods 

### 2.1. Biochar Preparation

**Figure 1:**
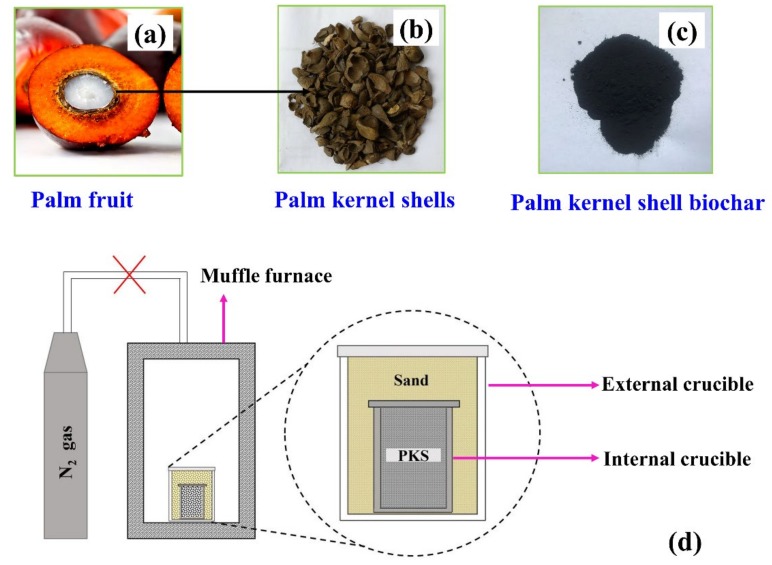
(**a**), (**b**), and (**c**) represent palm fruit, palm kernel shell (PKS), and palm kernel shell biochar (PKSBC), respectively. (**d**) Experimental configuration for biochar preparation.

Biochar was synthesized from palm kernel shells obtained from a palm-oil-producing dumpsite in Kukurantumi, Ghana. The palm kernel shell was washed with distilled water to remove impurities. Next, it was dried in an oven and later ground into smaller particles using a nut grinder to obtain a uniform particle size; the ground sample was sieved using a 200-mesh sieve. Forty grams of palm kernel shell powder (particle size < 0.074 mm) was heated in a muffle furnace to 900 °C in ambient air at a heating rate of 10 °C/min, and was held at this temperature for 1 h and allowed to cool down naturally to room temperature overnight. The experimental setup for this carbonization process is shown in Figure 1d. The relevance of sand in the external crucible is to reduce the diffusion of air (O_2_) into the inner crucible to prevent the oxidation of the precursor during heat treatment (Figure 1d). The resulting biochar is oven-dried at 120 °C for 24 h, after which it is bagged in plastic bags for further use.

### 2.2. Preparation of Mg-PKSBC Hydrogen Storage Material

Mg powder (particle size < 0.0074 mm, purity = 99.0 wt.%) purchased from Tianjin Ruijinte Chemical Company, China, was ball milled with different quantities of palm kernel shell biochar under an argon atmosphere in an ND7 model planetary ball-mill (Nanda Tianzun Instrument Company, China). Ball mill vail charging and sample handling were conducted in an argon-filled glove box (Etelux Lab2000). The milling conditions in this investigation were set as follows: ball to sample weight ratio 47:1, milling speed 180 r/min, milling time 1 h, and Mg powder:PKSBC ratios of 8:2, 9:1, and 9.5:5. The hydriding process was carried out on an automatically controlled isothermal absorption instrument (PCTPro-2000, Setaram France) at 360 °C and hydrogen pressure range of 0 MPa to 2.5 MPa. For comparison, pure magnesium powder was milled and hydrided under the same experimental conditions as the synthesized composites. Retrieved samples after ball milling and hydriding were denoted as 100*-x*Mg*x*PKSBC and H100*-x*Mg*x*PKSBC, respectively, where 100 represents the total weight percent of the composite and *x* represents the weight percent (wt.%) of PKSBC; thus 5 wt.%, 10 wt.%, and 20 wt.% were used.

### 2.3. Characterization 

XRD was performed on a Rigaku D/Max-rB X-ray diffraction instrument at a scanning speed of 8 deg/min and in steps of 0.02°. The thermal analysis of the material was carried out on a Setaram Sensys Evo DSC1 at an argon flow rate of 80 mL/min. Raman spectra were recorded on a Horiba (XploRA) spectrometer. The source radiation was a laser operating at a wavelength of 514 nm and power of 25 mW. The hydriding process was carried out on an automatically controlled high pressure isothermal adsorption instrument (PCTPro-2000, Setaram, France). The morphology of the materials was explored using a Japan Hitachi S-4800 scanning electron microscope with an EDS attached. Thermogravimetric (TG) and derivative thermogravimetric (DTG) analyses were conducted on an NETZSCH STA 409 PC/PG thermal analysis device at a heating rate of 10 °C/min in an N_2_ atmosphere. 

Textural characterization was conducted on micrometrics ASAP 2020 V4.02 at 77 K using N_2_ gas as the source gas. Prior to the adsorption measurements, the sample of activated carbon was outgassed under vacuum at 573 K overnight to remove any adsorbed moisture and gases. The relative pressure point (*P/Po*) range corresponding to the linear region in N_2_ adsorption isotherm data was used to calculate the specific surface area (*S*_BET_) by applying the Brunauer–Emmett–Teller (BET) equation. The total pore volume (*V*_tot_) was determined by the quantity of absorbed liquid nitrogen (volume) at a *P/Po* of 0.99. The micropore volume was determined by the t-plot method, a plot of thickness (*t*) against relative pressures (*P/Po*). The mesopore volume was calculated as the difference between the total pore volume and the t-plot micropore volume. From the formula 4*V*_tot_/*S*_BET_, the average pore size was calculated. The pore size distribution curves were determined by the density functional theory (DFT) method. FTIR analysis was performed on a Nicolet iS50 FTIR spectrometer.

## 3. Results and Discussions

### 3.1. Characterization of Prepared Palm Kernel Shell Biochar

**Figure 2:**
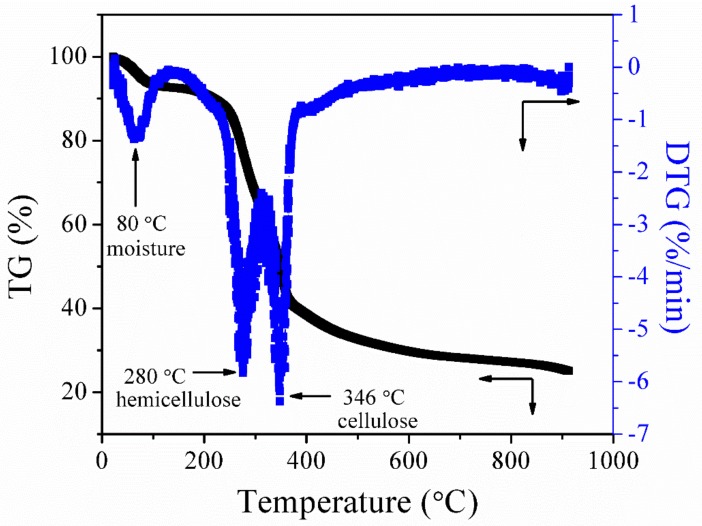
Thermogravimetric (TG)/derivative thermogravimetric (DTG) curves of palm kernel shell at a heating rate of 10 °C/min in a nitrogen atmosphere.

Figure 2 presents the thermal decomposition behavior (a function of mass loss with respect to increasing temperature) of raw palm kernel shell in the flow of nitrogen. The thermal degradation of palm kernel shell occurs by the breakdown of cellulose, hemicellulose, and lignin as the temperature increases from 25 °C to 900 °C. The peak appearing at 80 °C represents the elimination of the physically absorbed water. The second peak that appears at 280 °C signifies the breakdown of hemicellulose and the third peak at 346 °C represents the decomposition of cellulose [34]. After the third peak, the DTG curve flattens as the temperature increases to a higher temperature; this might be attributed to the gradual breakdown of lignin. Lignin in lignocellulose biomass is the last organic constituent to be broken down to produce char. It has also been discovered by other researchers that lignin decomposition occurs without observable peaks on the DTG curve [35].

**Figure 3:**
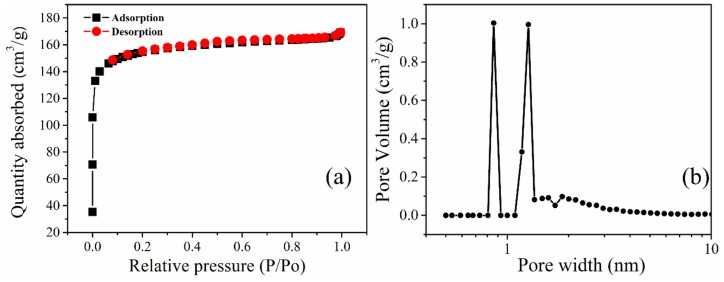
Nitrogen adsorption-desorption isotherm of PKSBC at 77 K (**a**) and pore size distribution curves of PKSBC (**b**).

Figure 3a,b and Table 1 show the porosimetry and pore size distribution (PSD) of the biochar prepared. The nitrogen adsorption-desorption isotherm at 77 K shown in Figure 3a shows a typical type I according to the IUPAC classification of porous materials [36]. It can also be observed that there is an absence of the hysteresis loop. From the adsorption curve, the steep rise in the amount of nitrogen gas absorbed at even a relatively low pressure signifies the presence of micropores. 

The presence of mesopores is confirmed by a steep rise in the plateau region. However, in this case, the plateau remains relatively flat even at higher pressure, signifying that there is a smaller amount of mesopores in the biochar. From the PSD curve (measured using the DFT method, assuming a split pore geometry for micropores based on N_2_ adsorption data), it can be observed that the biochar is predominantly microporous with the majority of pores in PKSBC having sizes less than 2 nm. The calculated total pore volume, micropore, and mesopore volume of 0.26 cm^3^g^−1^, 0.19 cm^3^g^−1^, and 0.07 cm^3^g^−1^, respectively, are shown in Table 1.

**Figure 4:**
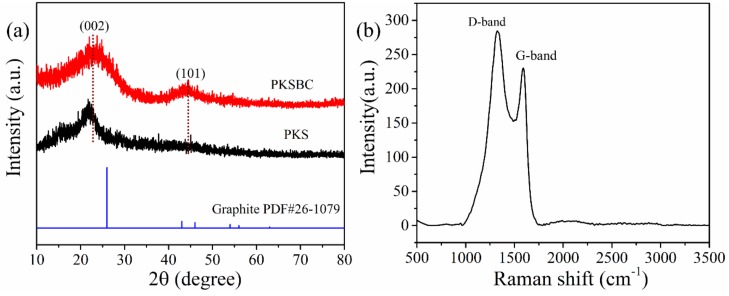
XRD patterns of PKS and PKSBC (**a**) and Raman spectra of PKSBC (**b**).

The XRD pattern of the biochar prepared at 900 °C and palm kernel shell is shown in Figure 4a. The presence of two broad diffraction peaks located at 2θ = 20°–30° and 40–50° reveals the presence of an amorphous structure as a result of disorderly stacked up carbon rings. When comparing graphite to PKSBC, it can be seen that the former possesses two distinct peaks of high crystallinity. The broad peaks that range from 20°–30° and 40°–50° correspond to (002) and (101) planes of the turbostratic form of carbon. Raw palm kernel shell, on the other hand, possesses only one diffraction peak at a 2θ value of 22°, indicating a weak crystalline nature of the sample. From the XRD patterns it can be observed that there is a slight shift in the (002) diffraction peak to higher diffraction angles. This peak shift can be attributed to the decreased interlayer spacing of the adjacent graphene stack layers as the carbonization occurs at a higher temperature [37]. It can also be noted that there is the appearance of a second peak, the (101) peak, which signifies structural rearrangement and an increased ordering of the graphene layers in the biochar [38]. According to [39], Raman spectroscopy is a useful characterization technique which is employed to analyze and detect the structural units of biomass chars. The Raman spectrum of PKSBC is shown in Figure 4b. PKSBC shows two distinct peaks located around 1324 cm^−1^ (D-band) and 1591 cm^−1^ (G-band) which can be attributed to the in-plane vibration of sp^2^-carbon structures with structural defects and in-plane vibrations of sp^2^-bonded graphitic structures, respectively [40]. In this case, PKSBC contains a large portion of amorphous carbon structures, consequently resulting in an I_D_/I_G_ (where I_D_/I_G_ represents the relative intensity of the D-band and G-band) value of 1.23, which also agrees with the 1.20 I_D_/I_G_ value of beech wood char synthesized in [40]. Comparing the I_D_/I_G_ value of PKSBC to that of pure graphite as reported by Xing et al. [41] to be 0.28, it can be inferred that PKSBC is amorphous.

**Figure 5:**
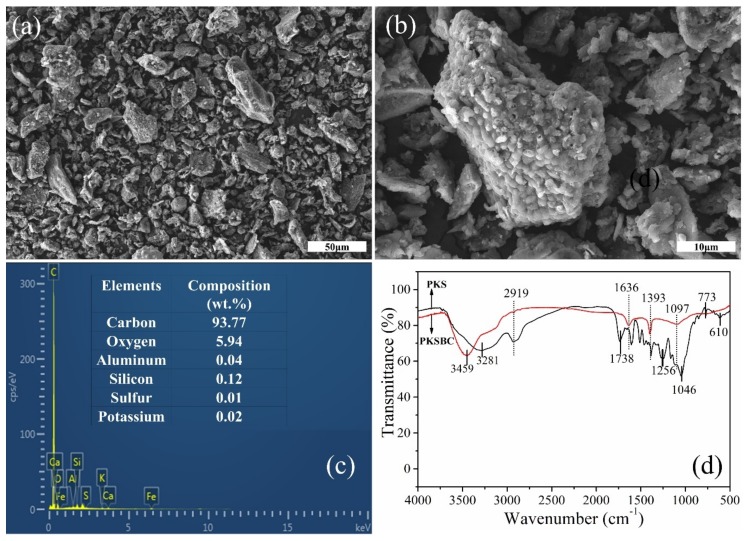
SEM images of PKSBC at different magnifications of 16k (**a**) and 60k (**b**), elemental composition of PKSBC (**c**), and FTIR spectrum of PKS and PKSBC (**d**).

In order to explore the surface morphology of the biochar, scanning electron microscope was employed to clearly observe it, as seen in Figure 5a,b. As shown in the figure, there are pores of various widths present on the particle surface, which can be attributed to the escaping of volatiles and other pyrolysis gases, such as hydrogen, CO, and CH_4_, from the precursor as the temperature increases. It will not be absurd to mention that there might be some nanosized pore network inside the individual particles of biochar. The elemental composition of PKSBC determined by an EDS is shown in Figure 5c, which reveals that the sample mainly contained carbon and oxygen, with elements Al, Si, K, and S present in trace quantities.

The surface functionalities which signify the chemical character of the biochar surface were characterized by FTIR. As seen in Figure 5d, the broad peak at around 3459 cm^−1^ and 3281 cm^−1^ can be assigned to the hydrogen-bonded hydroxyl functional group, showing the presence of water molecules in the raw sample and the biochar. After carbonization at 900 °C, the amount of various functional groups reduces, as shown in the FTIR spectrum. There is a complete disappearance of the 2919 cm^−1^ band (which can be attributed to a C-H stretching vibration) from the biochar, showing that hydrogen was eliminated during the carbonization process [42]. As indicated on the spectrum of PKS, the functional groups at band spot 1738 cm^−1^ (C=O stretching vibration of carboxyl group), 1393 cm^−1^ (C-H deformation bond), 1256 cm^−1^ (C-C skeletal vibration), 1046 cm^−1^ (C-O stretching bond of phenols), and 610 cm^−1^ (out of plane C-H blending with different degrees of substitution) either disappear or show a reduction in absorbance, leaving only two peaks at 1636 cm^−1^ (C=C stretching vibration) and 1393 cm^−1^ (CH_2_ and CH_3_ bending vibrations) as well as a diffused minor peak at 1097cm^−1^ (C-O stretching bond of the phenolic, carboxylic, and ester functional group) [43,44]. The raw sample shows a series of bands from 924 cm^−1^ to 500 cm^−1^ (representing some aromatics and C-H bending [45]) which clearly flattens out after the carbonization process. 

### 3.2. Crystal Structure of Materials

**Figure 6:**
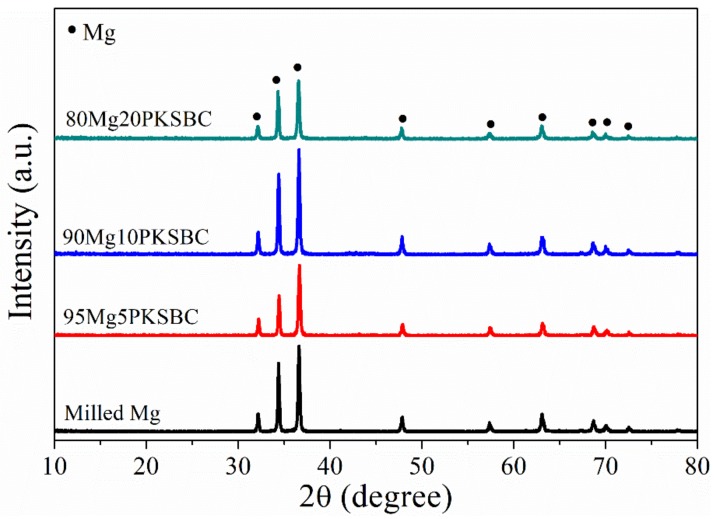
XRD patterns of ball milled Mg/PKSBC composite for 1h at a milling frequency of 30 Hz.

The XRD patterns of milled Mg and Mg-PKSBC composites, which predominantly consist of Mg phases, are presented in Figure 6. The diffraction peak of carbon is not apparent, which means the carbon from PKSBC is in order in a very short range, signifying it is amorphous. In the composites, the XRD diffraction of carbon is so weak as to be negligible, compared with that of β-MgH_2_ or Mg, whose crystal grains are much bigger. To evaluate the effect of ball milling pure Mg with PKSBC on the crystallite size of Mg, the Scherer equation [21] was employed to calculate the changes in crystallite size as the PKSBC content was incorporated into the milling process. The calculated average crystallite sizes (calculated by considering the major diffraction peaks of Mg 100, 002, and 101) for milled Mg and 80Mg20PKSBC were 45 nm and 40 nm, respectively. From this, it is sound to propose that PKSBC acts as a milling aid to an extent. Additionally, there was a slight reduction in the intensity of the major peaks of Mg when the PKSBC content increased to 20 wt.%. 

**Figure 7:**
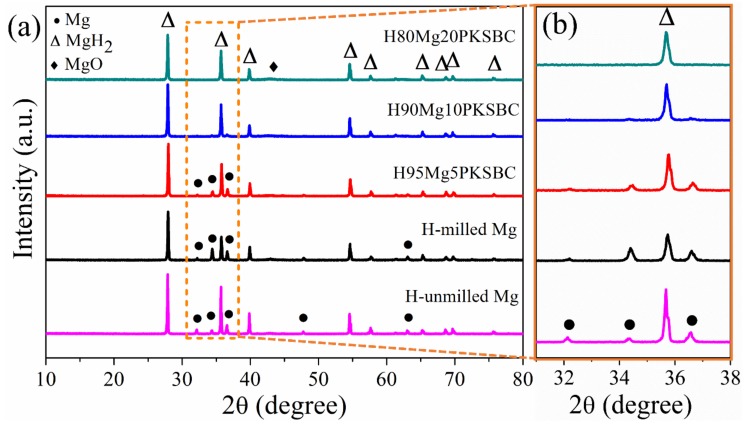
XRD patterns of (**a**) hydrided Mg/PKSBC at 360 °C and (**b**) enlarged rectangle in (a).

Figure 7a presents the XRD patterns of hydrided Mg/PKSBC at 360 °C. It shows that β-MgH_2_ is formed after high-pressure hydrogen absorption; from the XRD patterns of H95MgPKSBC and H90MgPKSBC it can be observed that Mg is present after the hydrogenation process. From these patterns it can clearly be seen that the amount of Mg phases reduces as the content of PKSBC increases from 5 wt.% to 20 wt.%. H80Mg20PKSBC patterns reveal the absence of Mg after hydrogenation, as shown in Figure 7b. However, MgO is detected when the PKSBC content is 20 wt.%, which can be attributed to the reaction between Mg and the oxygen-containing functional groups during the hydrogenation process at 360 °C. 

### 3.3. Effect of PKSBC Addition on the Preparation of Mg-Based Hydrogen Storage Materials

**Figure 8:**
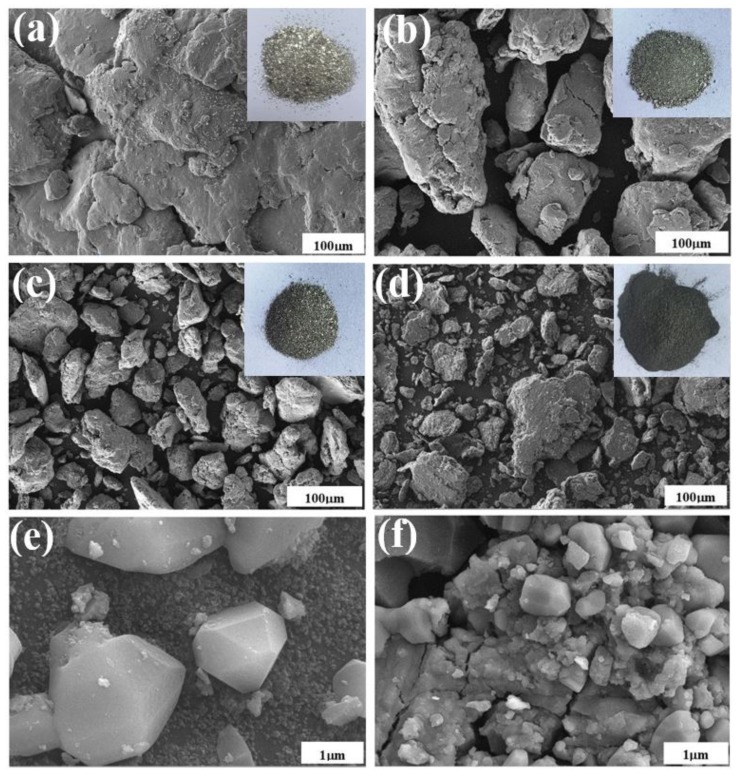
SEM images of milled Mg with insets of camera images (**a**), 95Mg5PKSBC (**b**), 90Mg10PKSBC (**c**), 80Mg20PKSBC (**d**), SEM images of H-milled Mg (**e**), and H80Mg20PKSBC (**f**) prepared at 360 °C.

The morphology of Mg-PKSBC after ball milling was examined by SEM, and the particles of the as-milled magnesium powders were found to be in the range of several hundreds of μm. Particles obtained after ball milling were much finer, being in the range 20 μm to 100 μm, with particles of magnesium adhering to each other to form a larger particle. Inset images from Figure 8a–d show decreasing agglomeration effects on magnesium particles after ball milling as PKSBC content is increased from 5 wt.% to 20 wt.%. The Mg/C composite containing 20 wt.% of PKSBC shows high material dispersion, as well as a reduced and a more uniform Mg particle size. From this observation, it can be accepted that PKSBC in the right amount acts as a dispersing agent. 

In addition, the lattice fringes of magnesium particles (Figure 8e,f) present in the hydrogen storage with PKSBC can be seen to be very smooth, while the sample without PKSBC has magnesium particles with sharp edges. When comparing the sizes of nucleated Mg phases present in the prepared hydrogen storage materials, the carbon-doped material depicts a smaller Mg phase size in contrast to the undoped sample.

### 3.4. Effect of PKSBC Addition on the Hydrogen Sorption Performance of Mg-Based Hydrogen Storage Materials

**Figure 9:**
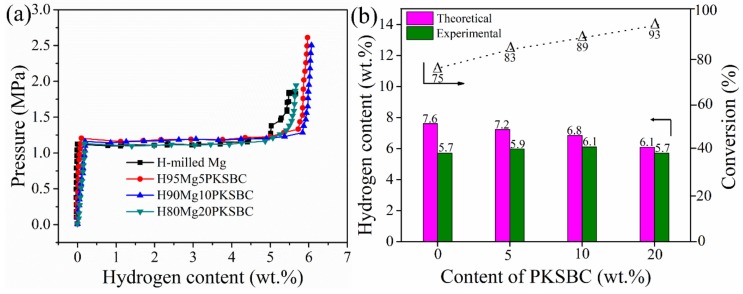
*p-c-*T curves of milled Mg and Mg-PKSBC composites (**a**) and bar chart comparison of theoretical, and experimental hydrogen capacity and conversion of Mg into MgH_2_ against PKSBC content (**b**).

During hydrogenation of magnesium particles at a constant temperature there exists a region of constant hydrogen pressure as the hydride is formed which is termed the plateau pressure region. Figure 9a shows the pressure-composition isotherm (*p-c*-T) curves of hydrogenated milled Mg and Mg/PKSBC at 360 °C. When comparing the hydrogen content of milled magnesium and 80Mg20PKSBC after hydriding at 360 °C, it can be observed that both samples have the same content of hydrogen. From theory it is known that reducing the content of magnesium decreases the amount of hydrogen absorbed. In this sense, the effect of the PKSBC can be appreciated in terms of reducing the particle size of the magnesium particles for near-complete hydrogenation of magnesium phases. Fully converting magnesium into magnesium hydride is usually impractical, even at very high temperatures and pressures. It has been revealed that hydrogen absorption declines when the hydride nuclei start to coalesce on the magnesium surface to form a compact hydride layer [46] and that the hydrogenation reaction diminishes completely when the hydride layer exceeds 30–50 µm. Further hydrogenation of magnesium is aborted due to kinetic factors. By contrast, hydrogenation of magnesium powder can be completed when the average particle radius is smaller than the width of the “blocking” hydride layer. Thus, to allow complete hydrogenation of magnesium, the powder radius should be less than 50 µm [47]. SEM images presented in Figure 8d show magnesium particles of an average size of less than 50 µm; this is one of the reasons why the conversion of magnesium into magnesium hydride in H80Mg20PKSBC is about 93%. It can also be noted that as the content of PKSBC increases, the conversion of Mg into MgH_2_ also increases; this can be seen in Figure 9b. The theoretical hydrogen storage capacity is calculated as the product of the Mg weight percent and hydrogen content of MgH_2_ (7.6 wt.% [48]). Complete hydriding of magnesium particles depends on both particle size and grain size; these two are inseparable. This observation cannot be applied to milled magnesium because of the relatively bigger particles due to agglomeration. The increase in the conversion of Mg into MgH_2_ may be partly due to the fact that the addition of PKSBC reduces the adhesion and agglomeration of Mg particles. These fine particles have a large surface area per unit volume which can enhance the hydrogen diffusion and hydride phase formation, which is therefore of benefit to the sorption properties. 

**Figure 10:**
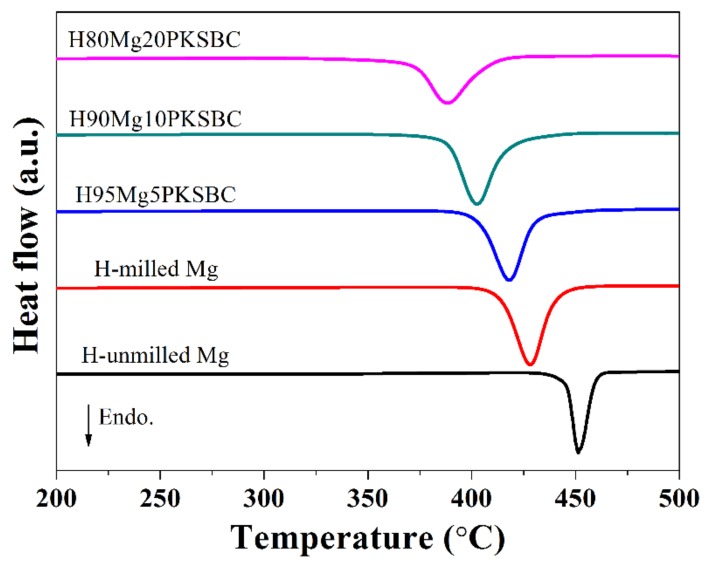
DSC curves of hydrogen storage materials at a heating rate of 10 °C/min.

From Figure 9b it can be concluded that the complete conversion of Mg to MgH_2_ is feasible if the right size is achieved before hydrogenation. Additionally, the presence of PKSBC can help in the physical adsorption of hydrogen atoms, which can be further absorbed chemically by Mg. The physical adsorption of hydrogen onto carbon is able to be supported by the literature [49], together with our previous report [21] in which the formation of C-H dangling bonds during the hydrogenation process occurred. Another way in which PKSBC is beneficial to hydrogen sorption is via its surface chemical character; the presence of oxygen-containing functional groups can positively influence the hydrogen sorption process [50]. In Figure 10, the effect of increasing the contents of PKSBC on the decomposition temperatures of prepared hydrogen storage materials is presented.

It is evident that increasing the amount of PKSBC decreases the decomposition temperature of MgH_2_. There is a 40 °C reduction in the decomposition temperature when compared with H-milled Mg and even a 63.5 °C decrease when compared with pure hydrogenated unmilled Mg. This reduction in temperature can be attributed to the reduced particle size of MgH_2_, which is in line with other reports [51], and the moderate surface area of the Mg/PKSBC composite. In the literature [52] it has been shown that relatively high textural properties of carbon additives improve the desorption process of MgH_2_.

It is still a matter of debate as to the exact mechanism which can explain the enhancement of hydrogen sorption properties of magnesium caused by carbon. On this note, we agree more with Adelhelm et al. [53], with regard to the fact that carbon materials, in general, have a positive influence on the hydrogen sorption properties of magnesium.

## 4. Conclusions

In this work, waste palm kernel shell derived biochar (with moderate textural properties and surface functional groups) was synthesized using a simple, cheap, and safe carbonization process which can be used as an additive for preparing Mg-based hydrogen storage materials. The prepared biochar acts as a milling aid and dispersant agent during the Mg/C preparation process. The optimum content of PKSBC for high conversion of Mg into MgH_2_ was found to be 20 wt.%. The incorporation of 20 wt.% PKSBC into the ball milling process of magnesium was found to benefit the hydrogen sorption of Mg by increasing the conversion of Mg into MgH_2_ to 93% compared with milled MgH_2_ (75%); this incorporation also lead to a 40 °C decrease in the MgH_2_ decomposition temperature. 

## Figures and Tables

**Table 1:** Textural properties of PKSBC.

*S*_BET_ (m^2^/g)	*Dp* (nm)	*V*_tot_ (cm^3^g^−1^)	*V*_micro_ (cm^3^g^−1^)	*V*_meso_ (cm^3^g^−1^)
594.50	1.76	0.26	0.19	0.07

Legend: *S*_BET_, surface area calculated by the Brunauer–Emmett—Teller (BET method); *Dp*, average pore size diameter calculated by 4*V/S*_BET_; *V*_tot_, total pore volume at *P/Po* = 0.99; *V*_micro_, micropore volume calculated by t-plot method; *V*_meso_, mesopore volume calculated by *V*_tot_ – *V*_micro_.
